# Increased frequency of rare missense *PPP1R3B* variants among Danish patients with type 2 diabetes

**DOI:** 10.1371/journal.pone.0210114

**Published:** 2019-01-10

**Authors:** Robina Khan Niazi, Jihua Sun, Christian Theil Have, Mette Hollensted, Allan Linneberg, Oluf Pedersen, Jens Steen Nielsen, Jørgen Rungby, Niels Grarup, Torben Hansen, Anette Prior Gjesing

**Affiliations:** 1 Department of Bioinformatics and Biotechnology, International Islamic University, Islamabad, Pakistan; 2 BGI-Europe, Copenhagen, Denmark; 3 Novo Nordisk Foundation Center for Basic Metabolic Research, Section of Metabolic Genetics, Faculty of Health Sciences, University of Copenhagen, Copenhagen, Denmark; 4 Center for Clinical Research and Prevention, Bispebjerg and Frederiksberg Hospital, Copenhagen, Denmark; 5 Department of Clinical Medicine, Faculty of Health and Medical Sciences, University of Copenhagen, Copenhagen, Denmark; 6 The Department of Clinical Research, University of Southern Denmark, Odense, Denmark; 7 DD2, SDCO, Odense University Hospital, Odense, Denmark; 8 Bispebjerg Hospital, University of Copenhagen, Copenhagen, Denmark; Tongji Med College, HUST, CHINA

## Abstract

**Background:**

*PPP1R3B* has been suggested as a candidate gene for monogenic forms of diabetes as well as type 2 diabetes (T2D) due to its association with glycaemic trait and its biological role in glycogen synthesis.

**Objectives:**

To study if rare missense variants in *PPP1R3B* increase the risk of maturity onset diabetes of the young (MODY), T2D or affect measures of glucose metabolism.

**Method:**

Targeted resequencing of *PPP1R3B* was performed in 8,710 samples; MODY patients with unknown etiology (*n* = 54), newly diagnosed patients with T2D (*n* = 2,930) and population-based control individuals (*n* = 5,726, of whom *n* = 4,569 had normal glucose tolerance). All population-based sampled individuals were examined using an oral glucose tolerance test.

**Results:**

Among *n* = 396 carriers, we identified twenty-three *PPP1R3B* missense mutations, none of which segregated with MODY. The burden of likely deleterious *PPP1R3B* variants was significantly increased with a total of 17 carriers among patients with T2D (0.58% (95% CI: 0.36–0.93)) compared to 18 carriers among non-diabetic individuals (0.31% (95% CI: 0.20–0.49)), resulting in an increased risk of T2D (OR (95% CI) = 2.57 (1.14–5.79), *p* = 0.02 (age and sex adjusted)). Furthermore, carriers with diabetes had less abdominal fat and a higher serum concentration of LDL-cholesterol compared to patients with T2D without rare missense *PPP1R3B* variants. In addition, non-diabetic carriers had a higher birth weight compared to non-carriers.

**Conclusion:**

Rare missense *PPP1R3B* variants may predispose to T2D.

## Introduction

The prevalence of type 2 diabetes (T2D) is reaching epidemic proportions. Currently, T2D affects approximately 415 million adults, and by 2040, this number is estimated to reach 642 million [1]. Genetic predisposition is an important risk factor for T2D [2].

Several underlying mechanisms may be involved in the development of T2D such as an insufficient insulin production and the lack of adequate insulin response in target tissues [3]. The hepatic postprandial conversion of glucose into glycogen is an important pathway which contributes to the disposal of glucose from the blood. Several enzymes and regulatory proteins are involved in hepatic glycogen synthesis and breakdown, and defects in this machinery may therefore result in diabetes.

PPP1R3B is the regulatory subunit increasing the activity of protein phosphatase 1 (PP1) which activates glycogen synthase and inactivates glycogen phosphorylase which is the rate-limiting enzyme in glycogenolysis [4,5]. The genomic region including *PPP1R3B* shows linkage to both T2D and monogenic diabetes [6,7], and genome-wide association studies (GWASs) have investigated the association of common variants in *PPP1R3B* with glucose metabolism and found significant associations with both T2D [8] and glycaemic traits such as association with decreased levels of fasting plasma glucose [9,10], increased levels of fasting serum insulin [11] in addition to increased serum levels of high-density lipoprotein (HDL)- and low-density lipoprotein (LDL)-cholesterol [9]. Yet, the effect of rare *PPP1R3B* variants has not previously been investigated and we know that rare (minor allele frequency (MAF) <0.1%) coding variants in certain genes affects the risk of developing T2D [12].

Thus, to examine the putative association between diabetes and rare missense variants not previously explored in the literature, we sequenced *PPP1R3B* among MODY probands with an unknown etiology (MODYX), patients with T2D and well-phenotyped non-diabetic individuals with the intension to study 1) if rare likely pathogenic mutations are of importance for the genetic aetiology of MODY in a Danish subset of patients; 2) if *PPP1R3B* missense variants associate with increased risk of T2D and 3) if *PPP1R3B* missense mutations affect measures of glucose metabolism in individuals with normal glucose tolerance, pre-diabetes or T2D.

## Materials and methods

### Study subjects

Targeted resequencing was performed in: 1) MODYX patients (*n* = 54) as well as three family-members of one of the probands recruited from the outpatient clinic at Steno Diabetes Center, Copenhagen, Denmark; 2) the Danish population-based Inter99 study [13] comprising individuals without diabetes, including prediabetic (*n* = 1,157) and glucose tolerant individuals (*n* = 4,569) in whom glucose tolerance was determined based on an oral glucose tolerance test (OGTT) and 3) the DD2-cohort (T2D-cohort), consisting of newly-diagnosed patients with T2D (*n* = 2,930) [14]. The selection criteria for the MODYX patients were: 1) One family-member with diagnosis before 25 years of age; 2) Preserved beta-cell function (diet or OHA treatment or measurable s-C-peptide > 3 years after diabetes diagnosis); 3) Anti-GAD65 negative (if measured) and 4) No known mutations in *HNF4A*, *GCK*, *HNF1A*, *HNF1B* or *INS*. Prediabetic individuals included participants having either impaired fasting glucose (IFG) or impaired glucose tolerance (IGT) after a 2-hour OGTT according to diagnostic criteria by the world health organization (WHO) 1999 [15]. All patients with T2D were glutamic acid decarboxylase (GAD) antibody-negative and had a fasting serum C-peptide concentration > 150 pmol/l within 1.5 years from diabetes diagnosis (if available).

Prior to participation, written informed consent was obtained from all participants. The study design was in accordance with the ethical scientific principles of the Helsinki Declaration II and approved by The Scientific Ethics Committee of the Capital Region of Denmark (Inter99: KA-98155; Steno: KA-93033) and by the Danish National Ethical Committee on Health Research (DD2: S-20100082).

### Anthropometric and biochemical analysis

Body weight (kg) was measured to the nearest 0.1 kg on a digital scale, while height (cm) was measured in an upright position to the nearest 0.5 cm using a non-extendable linen tape with the participant wearing light indoor clothes and no shoes. Body mass index (BMI) was calculated as weight in kilograms divided by height in meters squared (kg/m^2^). Waist circumference (cm) was measured at the umbilical level on subjects in an upright position to the nearest 0.5 cm with a non-extendable linen tape according to WHO recommendation [16]. The waist-hip ratio was calculated as waist circumference (cm) divided by hip circumference (cm).

The Inter99 cohort: A standard 75 g OGTT was performed after a 12-hour overnight fast. Serum insulin and plasma glucose were measured in samples obtained at 0, 30, and 120 minutes during the OGTT. Serum insulin levels (excluding des-31,32 and intact proinsulin) were measured using the AutoDELFIA insulin kit (Perkin-Elmer, Wallac, Turku, Finland). Plasma glucose was analysed using a glucose oxidase method (Granutest; Merck, Darmstadt, Germany) [17]. Concentrations of serum triglycerides, HDL-cholesterol, LDL-cholesterol, and total cholesterol were analysed using enzymatic colorimetric methods (GPO-PAP and CHOD-PAP, Roche Molecular Biochemicals, Germany). Haemoglobin A1c (HbA1c) was measured using ion-exchange high performance liquid chromatography (normal reference range: 4.1–6.4%) [18]. A clinical description of participants can be found in S1 Table.

The DD2-cohort: Measures of BMI and routine laboratory measurements, such as fasting blood glucose, fasting serum C-peptide, GAD-antibody and C reactive protein (CRP), were extracted from the Danish Diabetes Database for Adults [19].

### Targeted resequencing

Targeted resequencing was performed using a solution-based target region capture and subsequent next generation sequencing (NGS) of the coding regions of 265 genes involved in the development of diabetes and obesity, including *PPP1R3B* [20]. The methods for DNA extraction, target region capture, and NGS have previously been extensively described [20]. The final captured DNA libraries were sequenced using the Illumina HiSeq2000 Analyzer as paired-end 90 bp reads (following the manufacturer’s standard cluster generation and sequencing protocols). All *PPP1R3B* coding regions were covered with a minimum mean depth of 30X and a mean depth of 171X. The variants located in *PPP1R3B* were annotated using Annovar [21] with variants annotated according to transcript NM_001201329.

The linkage disequilibrium (LD) structure between presently identified and previously investigated variants in *PPPP1R3B* (chr8:8993264–9008720) [22] was calculated using LDlink [23]. Two variants (p.S41R and p.G48E) from the present study were in high LD (D^1^> 0.8) with previously investigated variants.

### Microarray-chip genotyping

DNA from four diabetic family members and eight non-diabetic family members was genotyped using the MetaboChip array [24] on a HiScan system (Illumina, SanDiego, California), and genotypes were called using GenomeStudio software (version2011.1; Illumina). From these genotypes, we extracted the region surrounding the *PPP1R3B* variant and estimated the haplotypes and co-segregation within one MODYX family using MERLIN [25] (S3 Table).

### Statistical analysis

A gene-based association analysis was performed using missense variants restricted to MAF<0.1% based on the total number of samples studied. The statistical difference in carrier-frequency between cases and controls was calculated using chi-squared, logistic regression adjusted for sex and age as well as a kernel-based adaptive cluster (KBAC) test [26]. Differences in quantitative traits were analysed using a linear regression using additive genetic models adjusted for age and sex. Analyses were conducted using R software (version 3.2.3; R Foundation for Statistical Computing, Boston, MA, USA) except KBAC which was performed using rvtests [27]. *A p*-value < 0.05 was considered statistically significant.

## Results

Targeted resequencing of *PPP1R3B* was performed in 54 MODYX patients, 2,930 patients with T2D, 1,157 pre-diabetic participants and 4,567 glucose tolerant individuals. A total of 23 missense mutations were found among 396 carriers of whom eight individuals were carrying two variants (S2 Table).

Among the 54 MODYX patients, four heterozygous *PPP1R3B* variants were found (p.R263W, p.G218E, p.S41R and p.G48E). Two of these (p.S41R and p.G48E) were common, having a MAF > 1% (1.7% and 3.3%, respectively) and are therefore unlikely to be MODY-causing variants. The pathogenicity of the remaining two variants was evaluated using the Combined Annotation Dependent Depletion (CADD) score where a PHRED-scaled CADD score above 10 predicts pathogenicity in the top 10 percentile of all variants and a score above 20 predicts the top 1 percentile [28]. The G218E variant was found to have a CADD-score of 24.3, in addition to a low MAF of 0.0008% in Europeans [29]. However, the prevalence of this variant was 0.4% among South Asians [29], and is therefore unlikely to be pathogenic. The p.R263W variant having a CADD score of 33 and a MAF of 0.003% is possibly a causal variant. DNA was available for three additional family members with diabetes and sequencing showed that they were all carriers of the p.R263W variant. In order to further establish the causality of this variant, eight family-members without diabetes were genotyped, and haplotypes were generated. The haplotype containing the p.R263W was found in four non-diabetic family members, which indicates that this variant is unlikely to be the causal variant within the examined MODYX-family.

In the 2,930 patients with T2D and 5,726 population-based control individuals, two common variants (p.S41R and p.G48E) were found. These two variants have been captured by previous GWASs [22] having much larger statistical power than the present study and the effect of these variants was not investigated further.

The remaining variants were all rare (MAF <0.1%) and have not been captured by previous GWASs and these were further investigated in relation T2D. The overall burden of rare missense variants among patients with T2D compared to non-diabetic individuals showed that the prevalence of rare missense variants was 0.58% among 2,930 cases, 0.52% among pre-diabetic individuals and 0.26% among glucose tolerant individuals (Table 1). Thus, a statistical significant difference in prevalence was found between non-diabetic individuals and patients with T2D using a logistic regression adjusting for sex and age (OR (95% CI): 2.57 (1.14–5.79, *p* = 0.02), Table 1).

**Table 1:** Number of carriers of rare (MAF<0.1%) missense *PPP1R3B* variants in glucose tolerant individuals (NGT), pre-diabetic individuals (IFG/IGT) and patients with T2D.

	NGT	IFG/IGT	T2D patients	NGT versus T2D patients	NGT+ IFG/IGT versus T2D patients
**Non-carriers (n)**	4,557	1,151	2,913		
**Carriers (n)**	12	6	17		
**Prevalence (%)** (95% confidence interval)	0.26 (0.15–0.46)	0.52 (0.24–1.13)	0.58 (0.36–0.93)		
**Fishers exact** OR (95% CI): *p*-value				2.22 (1.06–4.65) *p* = 0.03	1.85 (0.95–3.60) *p* = 0.07
**Logistic regression** OR (95% CI): *p*-value				3.07 (1.24–7.74) *p* = 0.02	2.57 (1.14–5.79) *p* = 0.02
**Kbac**				*p* = 0.04	*p* = 0.04

Enrichment of coding non-synonymous *PPP1R3B* variants having a MAF < 0.1% was also observed using the http://www.type2diabetesgenetics.org, where 51 carriers out of 9,121 patients with diabetes were found in contrast to 40 carriers out of 9,335 non-diabetic individuals [30]. Yet, this enrichment was not statistically significant. However, when selecting only coding non-synonymous variants having a MAF < 0.1% classified as possibly damaging, this enrichment become further augmented with 14 carriers among patient with diabetes in contrast to six among control individuals [30].

The association between rare *PPP1R3B* missense variants and measures of glucose metabolism were examined among 4,569 glucose tolerant individuals of whom 12 were carriers, 1,157 pre-diabetic participants of whom six were carriers (Table 2) and 2,930 patients with T2D of whom 17 were carriers of rare *PPP1R3B* variants (Table 3).

**Table 2:** The effect of missense *PPP1R3B* variants with a MAF <0.1% on measures of metabolism in 4,569 glucose tolerant individuals (NGT) and 1,157 pre-diabetic participants.

	Glucose tolerant individuals (NGT)			Prediabetic participants (IFG/IGT)		
Trait	Non-carriers (*n* = 4,557)	Carriers (*n* = 12)	*p*-value	Non-carriers (*n* = 1,151)	Carriers (*n* = 6)	*p*-value
Sex (m/w)	2108/ 2449	5/7	NA	697/454	3/3	NA
BMI (kg/m^2^)	25.0 (22.7–27.7)	24.8 (22.5–26.7)	0.7	27.5 (24.6–30.6)	27.1 (22.3–29.0)	0.3
CRP	0.74 (0.33–1.72)	0.54 (0.35–0.85)	0.3	1.27 (0.57–2.76)	0.59 (0.42–0.89)	0.06
Waist-hip ratio	0.84 (0.78–0.90)	0.84 (0.81–0.87)	0.4	0.90 (0.83–0.95)	0.85 (0.78–0.88)	**0.05**
HbA1c (%)	**5.80 (5.50–6.00)**	**6.05 (5.98–6.30)**	**0.02**	5.90 (5.70–6.20)	5.65 (5.43–5.88)	0.07
Fasting p-glucose (mmol/l)	5.30 (5.00–5.60)	5.35 (5.10–5.53)	1.0	6.10 (5.70–6.40)	6.20 (5.73–6.45)	0.7
p-glucose 30min (mmol/l)	8.10 (7.10–9.10)	8.05 (7.60–8.55)	0.9	9.90 (9.00–10.9)	8.90 (8.45–10.6)	0.6
p-glucose 120min (mmol/l)	5.50 (4.70–6.30)	5.75 (5.00–6.45)	0.5	8.00 (6.30–8.80)	7.95 (5.58–8.08)	0.3
Fasting s-C-peptide (pmol/l)	499.0 (394.0–640.0)	488.0 (366.8–514.5.0)	0.2	667.5 (508.3–883.8)	699.0 (608.0–801.3)	0.7
s-C-peptide 30min (pmol/l)	1880 (1490–2360)	1610 (1438–2240)	0.3	1975 (1560–2338)	2155 (1833–2755)	0.2
s-C-peptide 120min (pmol/l)	1960 (1490–2490)	1640 (1465–2543)	0.5	2880 (2140–3720)	3070 (2500–3490)	0.7
Fasting s-Insulin (pmol/l)	31.00 (22.00–46.00)	30.50 (22.00–39.75)	0.1	44.5 (29.0–67.0)	38.5 (28.3–44.3)	0.5
s-Insulin 30min (pmol/l)	243.0 (176.0–346.0)	185.0 (161.0–229.5)	0.5	258.0 (176.0–382.0)	265.0 (163.8–495.3)	0.3
s-Insulin 120min (pmol/l)	136.0 (87.0–209.0)	99.0 (82.0–159.0)	0.5	254.0 (155.0–442.3)	201.0 131.8–374.5)	0.6
BIGTT-AIR	1680 (1351–2109)	1531 (1429–1940)	0.4	1437 (1099–1936)	1581 (1363–2672)	0.07
BIGTT-SI	10.3 (7.81–12.8)	10.2 (9.94–13.9)	0.2	6.06 (3.76–8.40)	5.75 (4.81–8.39)	0.5
HOMAIR	1.24 (0.86–1.85)	1.05 (0.86–1.69)	0.5	2.01 (1.29–2.99)	1.75 (1.36–1.89)	0.3
Matsuda index	8.57 (5.98–11.8)	8.93 (7.97–12.11)	0.4	5.30 (3.49–7.74)	6.22 (4.59–11.1)	0.4
Insulinogent index	25.9 (18.0–37.8)	17.6 (15.8–23.4)	0.1	21.1 (14.1–32.4)	23.1 (18.0–50.9)	0.3
s-LDLc (mmol/L)	3.35 (2.78–4.02)	3.85 (3.21–4.57)	0.2	3.64 (3.04–4.32)	4.28 (3.72–4.57)	0.3
s-total cholesterol (mmol/L)	5.30 (4.70–6.10)	5.75 (5.25–6.53)	0.2	5.70 (5.00–6.50)	6.35 (5.48–6.48)	0.6
s-HDLc (mmol/L)	1.41 (1.17–1.69)	1.47 (1.18–1.74)	0.7	1.30 (1.10–1.59)	1.31 (1.17–1.41)	0.9
s-triglyceride (mmol/L)	1.00 (0.70–1.40)	0.95 (0.75–1.43)	0.8	1.30 (1.00–2.00)	1.25 (1.03–1.40)	0.8
**Birth-weight (g)**	**3,400 (3050–3750)**	**3,750 (3400–4200)**	**0.03**	**3,400 (3050–3750)**	**3,900 (3700–4050)**	**0.04**

Data is presented as median and interquartile range. Traits were all q-transformed. Values in bold are significant *p*-values. BMI, body mass index; HDLc: HDL-cholesterol; LDLc: LDL-cholesterol.

**Table 3:** Quantitative trait analysis of rare missense *PPP1R3B* variants in *n* = 2,930 newly-diagnosed patients with T2D.

Trait	Non-carriers (*n* = 2,913) Median (IQR)	Carriers (*n* = 17) Median (IQR)	p-value (sex and age adjusted)
Sex (men/women)*	1664/1174	14/3	_
Age at examination (years)	61.0 (53.0–68.0)	55.0 (52.0–61.0)	_
Age at diagnosis (years)	60.0 (52.0–67.0)	53.5 (47.0–57.0)	0.6
BMI (Kg/m^2^)	30.6 (27.0–34.6)	29.7 (27.1–32.6)	0.8
Waist-hip ratio (cm)	0.98 (0.92–1.03)	0.97 (0.94–1.03)	**0.03**
s-triglycerides (mmol/L)	1.60 (1.10–2.40)	1.70 (1.20–1.80)	0.6
s-total cholesterol (mmol/L)	4.40 (3.70–5.10)	4.65 (4.05–5.93)	0.3
s-HDLc (mmol/L)	1.20 (1.00–1.40)	1.20 (1.03–1.38)	0.5
s-LDLc (mmol/L)	2.20 (1.80–2.90)	3.40 (2.60–3.90)	**0.006**

Traits were all q-transformed.

* not available in 75 individuals from DD2 (T2D-cohort).

Values in bold are significant *p*-value. BMI, body mass index; HDLc: HDL-cholesterol; IQR, interquartile range; LDLc: LDL-cholesterol.

HbA1c was slightly elevated among glucose tolerant *PPP1R3B* variant carriers (non-carriers: (median (interquartile range (IQR)): 5.80% (5.50–6.00); carriers: 6.05% (IQR: 5.98–6.30), *p* = 0.04) as well as measures of birth weight (non-carriers: 3,400g (IQR: 3,050–3,750), carriers: 3,750g (IQR: 3,400–4,200), *p* = 0.03). The latter trait was also significantly elevated among pre-diabetic individuals (non-carriers: 3,400g (IQR: 3,050–3,750); carriers: 3,900g (IQR: 3,700–4,050), *p* = 0.04) (Table 2). Measures of birth weight were unavailable among patients with T2D.

Waist-hip ratio was slightly lower in carriers of rare *PPP1R3B* missense variants among both pre-diabetic individuals (carriers: 0.85 (IQR: 0.78–0.88); non-carriers: 0.90 (IQR: 0.83–0.95), *p* = 0.05) and patients with T2D (carriers: 0.97 (IQR: 0.94–1.03); non-carriers: 0.98 (IQR: 0.92–1.03), *p* = 0.03). Also a significantly higher level of plasma LDL-cholesterol was found among diabetic carriers (3.40 mmol/L (IQR: 2.6–3.9)) of rare *PPP1R3B* missense variants compared to patients with T2D without such variants (2.20 mmol/L (IQR: 1.80–2.90), (*p* = 0.008)) (Tables 2 and 3). Additionally, the gender distribution among patients with T2D carrying rare *PPP1R3B* variant was skewed with only three women compared to 14 men.

## Discussion

Targeted resequencing of *PPP1R3B* among 54 MODYX probands did not reveal any likely pathogenic variants. In contrast, our investigation of a large number of deeply phenotyped patients with T2D and control individuals indicates that the presence of rare deleterious *PPP1R3B* variants increases the risk of developing T2D, associates with an elevated level of HbA1C, a decreased waist-hip ratio, an elevated birth weight and among patients with T2D, of whom the majority of carriers were men, increased concentrations of plasma LDL-cholesterol (S1 Fig).

**Fig 1:**
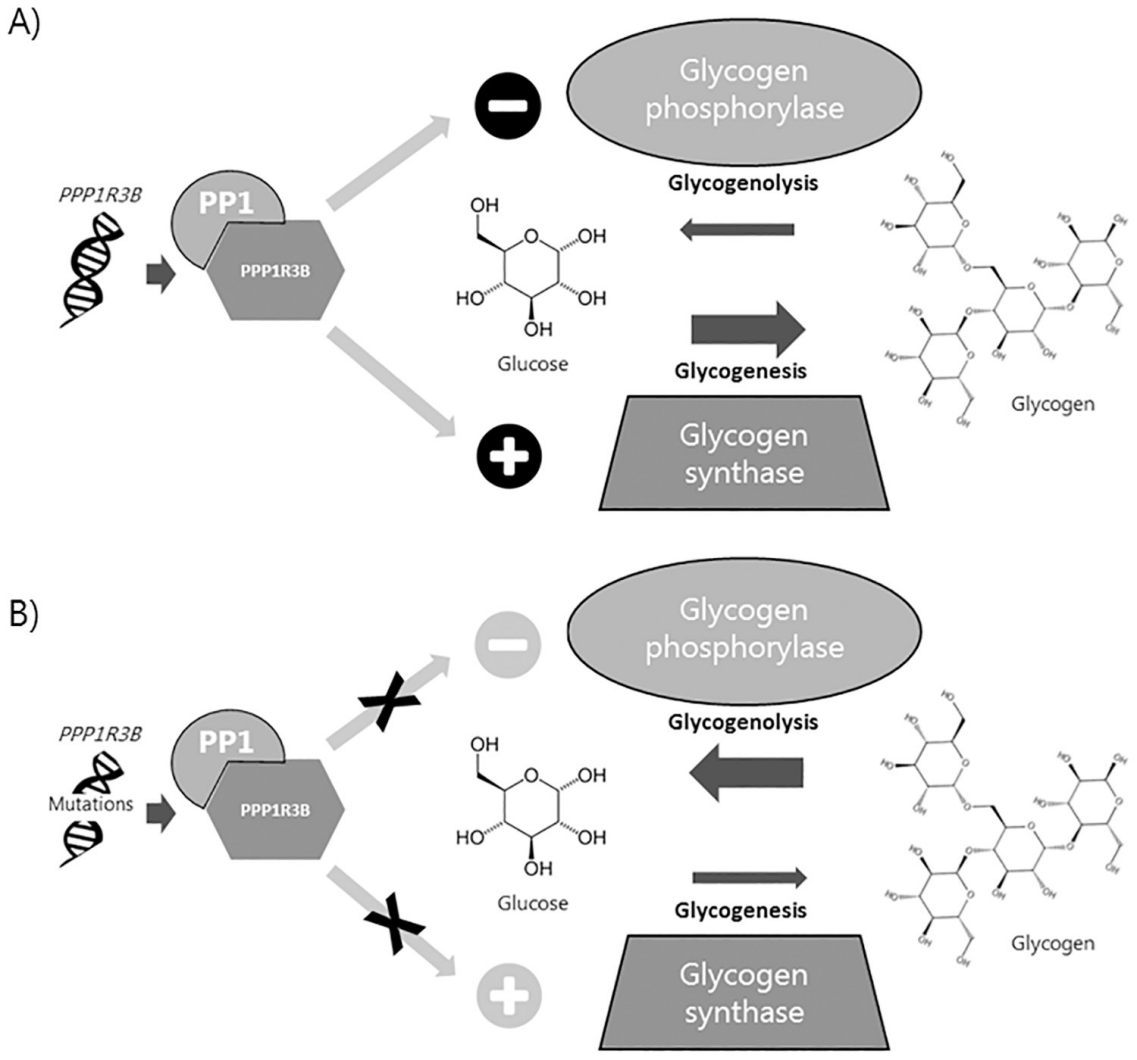
The hypothesised effect of *PPP1R3B* variants on glycogenesis and glycogenolysis. A) The PPP1R3B/protein phosphatase 1 (PP1) complex is an activator of glycogen synthase and an inhibitor of glycogen phosphorylase. Both functions of the PPP1R3B/PP1 complex will lead to an increase in glycogen due to increased conversion of glucose to glycogen and decreased breakdown of glycogen. B) Mutations in *PPP1R3B* may lead to reduced PPP1R3B/PP1 activity and consequently decreased activation of glycogen synthase and decreased inhibition of glycogen phosphorylase leading to a decreased level of glycogen.

In humans, *PPP1R3B* is expressed in both the liver and skeletal muscle. PPP1R3B is the regulatory subunit increasing the activity of PP1 which activates glycogen synthase, a key enzyme in glycogenesis, and inactivates glycogen phosphorylase which is the rate limiting enzyme in glycogenolysis [4,5]. Thus, the association observed between rare *PPP1R3B* variants and increased risk of T2D as well as elevated levels of HbA1c, could be caused by variants inactivating *PPP1R3B*, resulting in increased plasma glucose levels. This increase in plasma glucose would be due to both a lack of glycogen synthase activation and a lack of glycogen phosphatase inactivation–thus glycogen will not be formed from glucose, and the glycogen present will be catabolized (Fig 1).

We were unable to validate the findings of the present study, yet, online available data supports the observed enrichment of rare coding variants among patient with T2D. Elevated levels of HbA1c were only observed among glucose tolerant carriers, which may be a consequence of the larger number of glucose tolerant individuals compared to individuals with prediabetes or T2D. In addition, the strongest SNP association observed at the *PPP1R3B* locus from GWAS data based on nearly 90,000 individuals showed a significantly increased risk of T2D (*p* = 6.7*10^−11^) [8,31] further indicating that variation in the *PPP1R3B* locus do associate with variation in glycaemia.

Also our hypothesis is supported by results from the *PPP1R3B* liver-specific knockout mouse which also present with severely impaired hepatic glycogen synthase and decreased glycogen storage [32]. In relation to treatment of diabetes, glycogen phosphorylase contributes to hyperglycemia, and the interaction between glycogen phosphorylase and PP1 has been suggested as a potential novel anti-diabetic target by playing a role in allosteric regulation of glycogen synthesis [33,34]. Our study supports that optimizing the effect of the PPP1R3B/PP1 complex could be an anti-diabetic drug target.

The *PPP1R3B* is located on 8p23.1 which has been linked with T2D and monogenic diabetes [7,35]. Nevertheless, based on our findings, the linkage peak with MODY cannot be explained by the *PPP1R3B* variants found in the present study, and the association with T2D of the variants identified in the present study is insufficient to explain the T2D linkage peak. Within the 8p23.1 region there are several other candidate genes of interest such as *GAT4* and *BLK*. Therefore, these genes may be interesting candidate genes, potentially explaining the linkage peak on 8p23.1.

Serum LDL-cholesterol was also significantly elevated among diabetic carriers of *PPP1R3B* variants. In humans, the link between *PPP1R3B* and lipid metabolism was established by GWASs, including one conducted in >100,000 individuals of European descent [36]. This study demonstrated that the rs9987289 variant in the vicinity of *PPP1R3B*, which is located at an eQTL, affects plasma HDL-cholesterol, LDL-cholesterol, and total cholesterol with the allele increasing the expression of *PPP1R3B* lowering the levels of plasma lipids [36].

*PPP1R3B* variants such as rs4240624 have previously been associated not only with lipid concentrations but also with histologic non-alcoholic fatty liver disease (NAFLD) [9] which is characterized by increased hepatic triglyceride content. The variant associating with increased risk of NAFLD also associated with increased concentrations of LDL-cholesterol, HDL-cholesterol and decreased levels of glucose. Thus, variants in *PPP1R3B* may have pleiotropic effects on both glycaemic levels and lipid metabolism.

The association observed between *PPP1R3B* variants and increased birth weight has not previously been reported in the literature, and the immediate biological link between PPP1R3B and birth weight is unclear. An elevated level of maternal blood glucose is an important determinant of birth weight [37]; therefore, if the child has inherited the *PPP1R3B* variant from its mother, the child may have been exposed to higher levels of blood glucose, resulting in an increased birth weight.

The current study indicates that *PPP1R3B* mutation carriers have a slightly elevated level of plasma glucose, possibly due to the reduced activity of the glycogen synthase. This may explain why carriers of the *PPP1R3B* variants with T2D have a lower level of abdominal fat and no indication of reduced insulin production, as this form of diabetes may not only be a result of peripheral insulin resistance nor beta-cell deficiency but rather a dysfunctional hepatic glycogen metabolism. Diabetes is often considered as a disease characterized either by insulin deficiency or insulin resistance, primarily in skeletal muscle. However, the current study emphasises the possible hepatic influence on the development of diabetes. Our current findings may therefore contribute to deciphering the complex heterogeneity of T2D and consequently help improve future targeted diabetes treatment.

The inability to differentiate functional from non-functional variants is a limitation to this study. Protein stability information is available for amino acids 105–253 in *PPP1R3B*, thus, we are only able to estimate the *in silico* effect on protein stability of 11 of the 23 identified variants which is not sufficient for a valid sub-analysis of variants affecting protein stability. Therefore, we focussed on rare variants as this frequency spectrum has not previously been captured by GWASs, and these rare missense variants are more likely to be functional. However, benign rare variants may occur, and these variants will create noise which may mask the effect of functional rare variants. Despite this limitation, the present study was able to identify an association between carrying rare missense variants in *PPP1R3B* and the development of T2D.

### Conclusion

The present data indicates that *PPP1R3B* missense variants increase risk of developing T2D, possibly through altered glycogen synthase function and altered lipid metabolism.

## Supporting information

S1 Table Clinical description of participants. (DOCX)

S2 Table Identified missense variants in PPP1R3B among 4,569 glucose tolerant individuals (NGT), MODYX probands (n = 54), 1,157 prediabetic individuals (IFG/IGT) and 2,930 patients with T2D. (DOCX)

S3 Table Genotypes and family structure included in the haplotype analysis. (DOCX)

S1 Fig Schematic presentation of overall results. (TIF)

## Abbreviations

BMI: Body mass index
GAD: glutamic acid decarboxylase
HbA1c: Haemoglobin A1c
HDL: high-density lipoprotein
LD: linkage disequilibrium
LDL: low-density lipoprotein
MAF: Minor allele frequency
T2D: Type 2 diabetes

## References

[1] International Diabetes Federation IDA-tE (2017) www.diabetesatlas.org. 7th Edition ed.

[2] Poulsen P, Kyvik KO, Vaag A, Beck-Nielsen H (1999) Heritability of Type II (non-insulin-dependent) diabetes mellitus and abnormal glucose tolerance—a population-based twin study. Diabetologia 42: 139–145. 10064092 10.1007/s001250051131

[3] DeFronzo RA (1992) Pathogenesis of type 2 (non-insulin dependent) diabetes mellitus: a balanced overview. Diabetologia 35: 389–397. 1516769 10.1007/BF00401208

[4] Munro S, Cuthbertson DJR, Cunningham J, Sales M, Cohen PTW (2002) Human skeletal muscle expresses a glycogen-targeting subunit of PP1 that is identical to the insulin-sensitive glycogen-targeting subunit G(L) of liver. Diabetes 51: 591–598. 11872655 10.2337/diabetes.51.3.591

[5] Montori-Grau M, Guitart M, Lerin C, Andreu AL, Newgard CB, et al (2007) Expression and glycogenic effect of glycogen-targeting protein phosphatase 1 regulatory subunit GL in cultured human muscle. Biochem J 405: 107–113. 10.1042/BJ20061572 17555403 PMC1925244

[6] Dunn JS, Mlynarski WM, Pezzolesi MG, Borowiec M, Powers C, et al (2006) Examination of PPP1R3B as a candidate gene for the type 2 diabetes and MODY loci on chromosome 8p23. Ann Hum Genet 70: 587–593. 10.1111/j.1469-1809.2005.00248.x 16907705

[7] Kim SH, Ma X, Weremowicz S, Ercolino T, Powers C, et al (2004) Identification of a locus for maturity-onset diabetes of the young on chromosome 8p23. Diabetes 53: 1375–1384. 15111509 10.2337/diabetes.53.5.1375

[8] (2018) Type 2 Diabetes Knowledge Portal.

[9] Speliotes EK, Yerges-Armstrong LM, Wu J, Hernaez R, Kim LJ, et al (2011) Genome-wide association analysis identifies variants associated with nonalcoholic fatty liver disease that have distinct effects on metabolic traits. PLoS Genet 7: e1001324 10.1371/journal.pgen.1001324 21423719 PMC3053321

[10] Hayes MG, Urbanek M, Hivert MF, Armstrong LL, Morrison J, et al (2013) Identification of HKDC1 and BACE2 as genes influencing glycemic traits during pregnancy through genome-wide association studies. Diabetes 62: 3282–3291. 10.2337/db12-1692 23903356 PMC3749326

[11] Manning AK, Hivert MF, Scott RA, Grimsby JL, Bouatia-Naji N, et al (2012) A genome-wide approach accounting for body mass index identifies genetic variants influencing fasting glycemic traits and insulin resistance. Nat Genet 44: 659–669. 10.1038/ng.2274 22581228 PMC3613127

[12] Bonnefond A, Clement N, Fawcett K, Yengo L, Vaillant E, et al (2012) Rare MTNR1B variants impairing melatonin receptor 1B function contribute to type 2 diabetes. Nat Genet 44: 297–301. 10.1038/ng.1053 22286214 PMC3773908

[13] Jorgensen T, Borch-Johnsen K, Thomsen TF, Ibsen H, Glumer C, et al (2003) A randomized non-pharmacological intervention study for prevention of ischaemic heart disease: baseline results Inter99. Eur J Cardiovasc Prev Rehabil 10: 377–386. 10.1097/01.hjr.0000096541.30533.82 14663300

[14] Thomsen RW, Nielsen JS, Ulrichsen SP, Pedersen L, Hansen AM, et al (2012) The Danish Centre for Strategic Research in Type 2 Diabetes (DD2) study: Collection of baseline data from the first 580 patients. Clin Epidemiol 4: 43–48. 10.2147/CLEP.S30083 23071401 PMC3470453

[15] Organization WH (1999) World Health Organization Diagnosis and Classification of Diabetes Mellitus: Report of a WHO Consultation, in Part 1. World Health Organization, Geneva.

[16] deOnis M, Habicht JP (1996) Anthropometric reference data for international use: Recommendations from a World Health Organization Expert Committee. American Journal of Clinical Nutrition 64: 650–658. 10.1093/ajcn/64.4.650 8839517

[17] Glumer C, Jorgensen T, Borch-Johnsen K (2003) Prevalences of diabetes and impaired glucose regulation in a Danish population—The Inter99 study. Diabetes Care 26: 2335–2340. 12882858 10.2337/diacare.26.8.2335

[18] Lau C, Vistisen D, Toft U, Tetens I, Glumer C, et al (2011) The effects of adding group-based lifestyle counselling to individual counselling on changes in plasma glucose levels in a randomized controlled trial: the Inter99 study. Diabetes Metab 37: 546–552. 10.1016/j.diabet.2011.06.001 21900030

[19] Gedebjerg A, Almdal TP, Berencsi K, Rungby J, Nielsen JS, et al (2018) Prevalence of micro- and macrovascular diabetes complications at time of type 2 diabetes diagnosis and associated clinical characteristics: A cross-sectional baseline study of 6958 patients in the Danish DD2 cohort. J Diabetes Complications 32: 34–40. 10.1016/j.jdiacomp.2017.09.010 29107454

[20] Gao R, Liu YX, Gjesing AP, Hollensted M, Wan XZ, et al (2014) Evaluation of a target region capture sequencing platform using monogenic diabetes as a study-model. Bmc Genetics 15. 10.1186/1471-2156-15-13 PMC3943834 24476040

[21] Wang K, Li MY, Hakonarson H (2010) ANNOVAR: functional annotation of genetic variants from high-throughput sequencing data. Nucleic Acids Research 38. 10.1093/nar/gkq603 PMC2938201 20601685

[22] Scott RA, Scott LJ, Magi R, Marullo L, Gaulton KJ, et al (2017) An Expanded Genome-Wide Association Study of Type 2 Diabetes in Europeans. Diabetes 66: 2888–2902. 10.2337/db16-1253 28566273 PMC5652602

[23] Machiela MJ, Chanock SJ (2015) LDlink: a web-based application for exploring population-specific haplotype structure and linking correlated alleles of possible functional variants. Bioinformatics 31: 3555–3557. 10.1093/bioinformatics/btv402 26139635 PMC4626747

[24] Voight BF, Kang HM, Ding J, Palmer CD, Sidore C, et al (2012) The metabochip, a custom genotyping array for genetic studies of metabolic, cardiovascular, and anthropometric traits. PLoS Genet 8: e1002793 10.1371/journal.pgen.1002793 22876189 PMC3410907

[25] Abecasis GR, Cherny SS, Cookson WO, Cardon LR (2002) Merlin-rapid analysis of dense genetic maps using sparse gene flow trees. Nature Genetics 30: 97–101. 10.1038/ng786 11731797

[26] Liu DJ, Leal SM (2010) A novel adaptive method for the analysis of next-generation sequencing data to detect complex trait associations with rare variants due to gene main effects and interactions. PLoS Genet 6: e1001156 10.1371/journal.pgen.1001156 20976247 PMC2954824

[27] Zhan X, Hu Y, Li B, Abecasis GR, Liu DJ (2016) RVTESTS: an efficient and comprehensive tool for rare variant association analysis using sequence data. Bioinformatics 32: 1423–1426. 10.1093/bioinformatics/btw079 27153000 PMC4848408

[28] Kircher M, Witten DM, Jain P, O’Roak BJ, Cooper GM, et al (2014) A general framework for estimating the relative pathogenicity of human genetic variants. Nat Genet 46: 310–315. 10.1038/ng.2892 24487276 PMC3992975

[29] Lek M, Karczewski KJ, Minikel EV, Samocha KE, Banks E, et al (2016) Analysis of protein-coding genetic variation in 60,706 humans. Nature 536: 285–291. 10.1038/nature19057 27535533 PMC5018207

[30] (2018) Type 2 Diabetes Knowledge Portal.

[31] Mahajan A, Taliun D, Thurner M, Robertson NR, Torres JM, et al (2018) Fine-mapping type 2 diabetes loci to single-variant resolution using high-density imputation and islet-specific epigenome maps. Nature Genetics. 10.1038/s41588-018-0241-6 PMC6287706 30297969

[32] Mehta MB, Shewale SV, Sequeira RN, Millar JS, Hand NJ, et al (2017) Hepatic protein phosphatase 1 regulatory subunit 3B (Ppp1r3b) promotes hepatic glycogen synthesis and thereby regulates fasting energy homeostasis. J Biol Chem 292: 10444–10454. 10.1074/jbc.M116.766329 28473467 PMC5481556

[33] Zibrova D, Grempler R, Streicher R, Kauschke SG (2008) Inhibition of the interaction between protein phosphatase 1 glycogen-targeting subunit and glycogen phosphorylase increases glycogen synthesis in primary rat hepatocytes. Biochemical Journal 412: 359–366. 10.1042/BJ20071483 18298402

[34] Cohen P (2006) Timeline—The twentieth century struggle to decipher insulin signalling. Nature Reviews Molecular Cell Biology 7: 867–873. 10.1038/nrm2043 17057754

[35] Pezzolesi MG, Nam M, Nagase T, Klupa T, Dunn JS, et al (2004) Examination of candidate chromosomal regions for type 2 diabetes reveals a susceptibility locus on human chromosome 8p23.1. Diabetes 53: 486–491. 14747302 10.2337/diabetes.53.2.486

[36] Teslovich TM, Musunuru K, Smith AV, Edmondson AC, Stylianou IM, et al (2010) Biological, clinical and population relevance of 95 loci for blood lipids. Nature 466: 707–713. 10.1038/nature09270 20686565 PMC3039276

[37] Scholl TO, Sowers M, Chen X, Lenders C (2001) Maternal glucose concentration influences fetal growth, gestation, and pregnancy complications. Am J Epidemiol 154: 514–520. 11549556 10.1093/aje/154.6.514

