# Supplementary material for: Increased frequency of rare missense PPP1R3B variants among Danish patients with type 2 diabetes
Source: PLoS One. 2019 Jan 10;14(1):e0210114. doi: 10.1371/journal.pone.0210114 (PMC6328241; doi:10.1371/journal.pone.0210114)
Supplement: S1 Table — (DOCX) [file pone.0210114.s001.docx]

**Supporting information**

**S1 Table:** Clinical description of participants.

| **Trait** | **Glucose tolerant**  **(Inter99, *n*=4,569)**  **Median (IQR)** | **Pre-diabetes**  **(Inter99, *n*=1,157)**  **Median (IQR)** | **Patients with T2D**  **(*n*=2,930)**  **Median (IQR)** | **MODYX probands**  **(*n*=54)**  **Median (IQR)** |
| --- | --- | --- | --- | --- |
| **Sex** | 2,113/2456 | 700/457 | 1,678/1,177 | 26/ 28 |
| **Age (years)** | 45.0 (39.9- 50.1) | 49.9 (44.8- 55.0) | 61.0 (53.0- 68.0) | 19.0 (14.9-24.2) |
| **BMI (kg/m^2^)** | 25.0 (22.7- 27.7) | 27.5 (24.6- 30.6) | 30.6 (27.0- 34.6) | 22.2 (19.4- 27.7) |
| **Fasting plasma glucose (mmol/l)** | 5.30 (5.00- 5.60) | 6.10 (5.70- 6.40) | 7.11 (6.39-8.12) | 8.90 (6.40- 9.80) |
| **Fasting serum- C-peptide (pmol/l)** | 499.0 (394.0- 640.0) | 668.5 (508.8- 883.2) | 1,148 (870.4-1,502) | 485.2 (289.8- 631.2) |
| **s-triglyceride (mmol/L)** | 1.00 (0.70- 1.40) | 1.30 (1.00-1.98) | 1.60 (1.00- 2.40) | 1.75 (1.285- 2.18) |
| **s-total cholesterol (mmol/L)** | 5.30 (4.70- 6.10) | 5.70 (5.00- 6.50) | 4.40 (3.70-5.10) | 5.00 (4.20- 5.20) |

IQR, interquartile range
