# Supplementary material for: Increased frequency of rare missense PPP1R3B variants among Danish patients with type 2 diabetes
Source: PLoS One. 2019 Jan 10;14(1):e0210114. doi: 10.1371/journal.pone.0210114 (PMC6328241; doi:10.1371/journal.pone.0210114)
Supplement: S2 Table — (DOCX) [file pone.0210114.s002.docx]

**Supporting information**

**S2 Table:** Identified missense variants in *PPP1R3B* among 4,569 glucose tolerant individuals (NGT), MODYX probands (n=54), 1,157 prediabetic individuals (IFG/IGT) and 2,930 patients with T2D.

| **No** | **Position** | **Amino acid** | **T2D carriers**  **(het/hom)** | **MODYX**  **carriers (het/hom)** | **IFG/IGT**  **carriers**  **(het/hom)** | **NGT**  **carriers (het/hom)** | **MAF in EXAC**  **(All)** | **MAF in total cohort** | **Functionality prediction (CADD-score)** |
| --- | --- | --- | --- | --- | --- | --- | --- | --- | --- |
| **Common variants (MAF > 1%)** | | | | | | | | | |
| 6 | 8999039 | p.S41R | 61/0 | 1/0 | 34/0 | 128/1 | 1.7*10^-2^ | 1.3*10^-2^ | 12.3 |
| 7 | 8999019 | p.G48E | 48/0 | 1/0 | 12/0 | 73/0 | 3.3*10^-2^ | 8.1*10^-3^ | 11.6 |
| **Low frequency variants (0.1%>MAF<1%): none** | | | | | | | | | |
| **Rare variants (MAF < 0.1%)** | | | | | | | | | |
| 1 | 8999130 | p.N11S | 1/0 | 0/0 | 0/0 | 0/0 | 8.4*10^-6^ | 4.4*10^-5^ | 0.21 |
| 2 | 8999115 | p.S16Y | 1/0 | 0/0 | 0/0 | 0/0 | 0 | 4.4*10^-5^ | 12.3 |
| 3 | 8999110 | p.R18C | 1/0 | 0/0 | 1/0 | 1/0 | 1.7*10^-5^ | 1.3*10^-4^ | 3.1 |
| 4 | 8999109 | p.R18H | 1/0 | 0/0 | 0/0 | 0/0 | 1.0*10^-4^ | 4.4*10^-5^ | 21.8 |
| 5 | 8999070 | p.S31N | 4/0 | 0/0 | 1/0 | 0/0 | 0 | 2.2*10^-4^ | 13.6 |
| 8 | 8998991 | p.K57N | 0/0 | 0/0 | 0/0 | 1/0 | 0 | 4.4*10^-5^ | 11.4 |
| 9 | 8998897 | p.P89S | 1/0 | 0/0 | 0/0 | 0/0 | 8.2*10^-6^ | 4.4*10^-5^ | 7.3 |
| 10 | 8998840 | p.E108Q | 2/0 | 0/0 | 1/0 | 1/0 | 8.3*10^-5^ | 1.8*10^-4^ | 20.2 |
| 11 | 8998765 | p.V133I | 1/0 | 0/0 | 0/0 | 0/0 | 3.3*10^-5^ | 4.4*10^-5^ | 32.0 |
| 12 | 8998699 | p.A155S | 1/0 | 0/0 | 0/0 | 0/0 | 1.7*10^-5^ | 4.4*10^-5^ | 22.8 |
| 13 | 8998668 | p.T165M | 0/0 | 0/0 | 1/0 | 2/0 | 1.0*10^-4^ | 1.8*10^-4^ | 32.0 |
| 14 | 8998618 | p.D182N | 0/0 | 0/0 | 0/0 | 1/0 | 0 | 4.4*10^-5^ | 23.9 |
| 15 | 8998606 | p.G186S | 3/0 | 0/0 | 1/0 | 1/0 | 4.4*10^-3^ | 2.2*10^-4^ | 7.1 |
| 16 | 8998579 | p.D195N | 0/0 | 0/0 | 1/0 | 1/0 | 1.7*10^-5^ | 8.8*10^-5^ | 10.4 |
| 17 | 8998519 | p.E215K | 0/0 | 0/0 | 0/0 | 1/0 | 1.7*10^-5^ | 4.4*10^-5^ | 23.5 |
| 18 | 8998509 | p.G218E | 0/0 | 1/0 | 0/0 | 0/0 | 7.0*10^-4^ | 4.4*10^-5^ | 24.3 |
| 19 | 8998473 | p.Y230C | 0/0 | 0/0 | 0/0 | 1/0 | 1.7*10^-5^ | 8.8*10^-5^ | 26.4 |
| 20 | 8998459 | p.A235P | 0/0 | 0/0 | 0/0 | 1/0 | 0 | 4.4*10^-5^ | 23.0 |
| 21 | 8998456 | p.E236Q | 1/0 | 0/0 | 0/0 | 0/0 | 0 | 4.4*10^-5^ | 14.6 |
| 22 | 8998375 | p.R263W | 0/0 | 1/0 | 0/0 | 0/0 | 2.5*10^-5^ | 4.4*10^-5^ | 33 |
| 23 | 8998368 | p.S265F | 0/0 | 0/0 | 0/0 | 1/0 | 8.2*10^-6^ | 4.4*10^-5^ | 30.0 |

Het: heterozygous; Hom: homozygous
