# Supplementary material for: Increased frequency of rare missense PPP1R3B variants among Danish patients with type 2 diabetes
Source: PLoS One. 2019 Jan 10;14(1):e0210114. doi: 10.1371/journal.pone.0210114 (PMC6328241; doi:10.1371/journal.pone.0210114)
Supplement: S3 Table — (DOCX) [file pone.0210114.s003.docx]

S3 Table: Genotypes and family structure included in the haplotype analysis

HHID ID FA MO Sex Pheno rs2409113 rs9329175 rs1016512 rs17155132 rs2028806 rs2979243 rs6601286 rs330921 rs330944 rs435581 rs367543 rs6999694 rs11249905 rs17155334 rs3102078 rs2929466 rs2929292 rs2929290 rs2929459 rs2929313

1 4 102 101 2 2 A G A A A G C C G G A A A C G G A G A A A G C A G A A G A A A A A A A C A A G G

1 3 102 101 2 1 A G A A A G C C G G A A A C G G A G A A A G C A G A G G A A C A A A A A G A A G

1 1 102 101 1 2 G G G A A G C C A G A A C C A G A G G A A G C A G A A G C A C A G A C C A A G G

1 101 0 0 2 1 A G G A G G A C G G G A A C G G G G A A G G C A A A A G A A C A A A A C G A A G

1 6 1 21 2 1 G G G G G G A C A G G A C C A G A G G A A G C A G A A G C A C C G A A C G A A G

1 5 1 21 1 1 A G A A A G C C G G A A A C G G G G A A G G C A A A A G A A A A A A C C A A G G

1 7 22 4 1 1 G G G A A G C C G G G A C C G G A G G A A G A A G A A G C A C A G A C C A A G G

1 2 102 101 2 1 A G A A A G C C G G A A A C G G A A A A A G C A G A G G A A C A A A A A G A A G

1 8 22 4 1 1 G G G A A G C C G G G A C C G G A G G A A G A A G A A G C A C A G A C C A A G G

1 9 22 4 1 1 G G G A A G C C G G G A C C G G A A G A A A C A G G G G C A C A G A A C A A G G

1 10 22 4 1 2 A G A A A G C C G G A A A C G G G G A A G G C A A A A G A A A A A A C C A A G G

1 11 22 4 2 1 A G G A G G C C G G G A A C G G A A G A A A C A G G G G C A C A G A A C A A G G

1 12 22 4 2 2 A G A A A G C C G G A A A C G G G G A A G G C A A A A G A A A A A A C C A A G G
