# Supplementary figures and images for: Increased frequency of rare missense PPP1R3B variants among Danish patients with type 2 diabetes
Source: PLoS One. 2019 Jan 10;14(1):e0210114. doi: 10.1371/journal.pone.0210114 (PMC6328241; doi:10.1371/journal.pone.0210114)

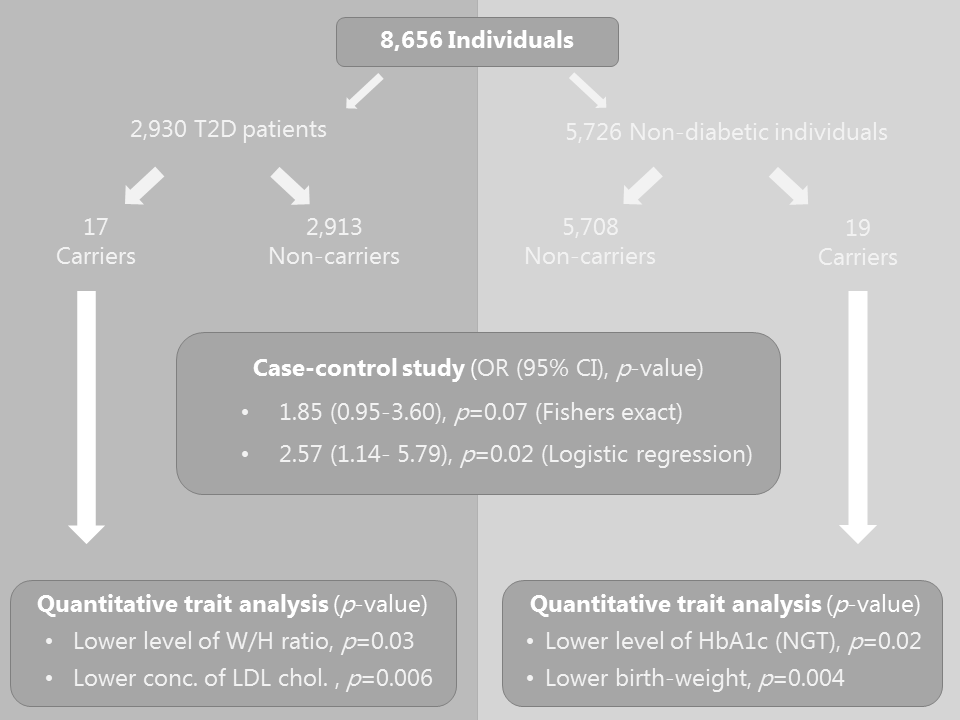

Supplement: S1 Fig — (TIF) [file pone.0210114.s004.tif]
